# Testican‐1‐MMP axis in tumour extracellular matrix (ECM) remodelling: interaction dynamics analysis and an *in silico* perspective

**DOI:** 10.1002/path.70073

**Published:** 2026-05-15

**Authors:** Sepideh Youssefi, Kiarash Saleki, Prerna Kadam, Amirreza Mazloomi, Abhik Mukherjee, Lodewijk V Dekker, Abdolrahman S Nateri

**Affiliations:** ^1^ Cancer Genetics & Stem Cell Group, The BioDiscovery Institute, School of Medicine University of Nottingham Nottingham UK; ^2^ School of Medicine, The Biodiscovery Institute, University of Nottingham Nottingham UK; ^3^ Student Research Committee Babol University of Medical Sciences Babol Iran; ^4^ Academic Unit of Translational Medical Sciences, School of Medicine University of Nottingham Nottingham UK; ^5^ School of Pharmacy, The Biodiscovery Institute University of Nottingham Nottingham UK

**Keywords:** Testican‐1 (SPOCK1), matrix metalloproteinases (MMPs), extracellular matrix (ECM) remodelling, tumour microenvironment, computational structural immunology

## Abstract

The extracellular matrix (ECM) plays a pivotal role in facilitating tumour development, invasiveness, metastasis, and immunoevasive processes through dynamic ECM remodelling processes. Testican‐1 (SPOCK1), an excretory matricellular proteoglycan, is suggested to exert a role in the facilitation of ECM remodelling processes through interacting with matrix metalloproteases (MMPs) and even its less known forms. The structural mechanisms of interactions between testican‐1 and MMPs were studied, and their roles in tumour‐promotion pathway processes were also examined using a computational approach and immunofluorescence validated by colocalisation technique analysis. A computational analysis using docking, molecular dynamics (MD), and systems biology analysis was employed. HDock and GROMACS were chosen to analyse binding affinity and testican‐1 stability with 28 different MMPs. H‐bond, free energy, and root mean square fluctuation (RMSF) analyses were performed to confirm the interactions in the testican‐1–MMP complexes. The systems biology toolkit implemented in this study consisted of STRING, BioGRID, and Cytoscape, which were employed for testican‐1 interaction network and pathway analysis. Kaplan–Meier survival analysis using the GEPIA2 tool was utilised to correlate *SPOCK1* gene expression and clinical survival measures for various malignancies. The docking analysis showed robust interactions between testican‐1 and MMP23, MMP25, and MMP28. Additionally, testican‐1–MMP complexes were confirmed to form stable interfaces based on comprehensive MD analysis, suggesting solid binding interfaces with the MMP‐unique domain of testican‐1. Our systems biology experiment indicated testican‐1 as a central hub for interactions between immunoevasive and ECM remodelling processes. SPOCK expression was also shown to correlate with significant survival measures for different malignancies, revealing clinical implications in cancer. The testican‐1–MMP computational analysis suggests testican‐1 plays a pivotal role as a therapeutic target for a wide range of malignancies. SPOCK–MMP interactions could be targeted to interrupt tumour‐promoting processes by arresting dynamic changes in the ECM, thereby improving patient survival. © 2026 The Author(s). *The Journal of Pathology* published by John Wiley & Sons Ltd on behalf of The Pathological Society of Great Britain and Ireland.

## Introduction

Among the extracellular matrix (ECM) regulators, the glycoprotein testican‐1 (SPOCK1) has been found to be a multifunctional modulator of the stability of the matrix and cellular functions. At the molecular level, the presence of the signal peptide, the unique domain, the follistatin‐like domain, the thyroglobulin domain, and the unique domain in the protein enables the protein to bind MMPs and other matrix proteins. Additionally, glycosylation and phosphorylation at the post‐translational level regulate the protein function and its role in cancer. Based on the regulation of matrix metalloproteinases (MMPs) and the matrix structure, the protein has the potential to be utilised as both a marker and the therapeutic agent in metastatic cancer [1, 2, 3].

MMPs denote a group of zinc‐dependent endopeptidases with pivotal roles in ECM remodelling in physiological and pathological mechanisms [4]. Physiological mechanisms include normal developmental processes, tissue repair, and angiogenesis. On the contrary, their overexpression or lack of regulation in cancer has been linked to tumour invasion, angiogenesis, and distant metastasis [5, 6, 7]. As there are different subtypes with a diverse range of substrates and domains, MMPs are subdivided based on specific criteria (supplementary material, Table S1). Within the group of MMPs are collagenases, such as MMP1 and MMP8. There are also gelatinases, which include MMP2 and MMP9. The stromelysin subfamily is composed of MMP3 and MMP10. MMPs that attach to the cell membrane are called membrane‐type matrix metalloproteinases, such as MMP14 [7, 8]. Among the MMPs, MMP2 and MMP9 play key roles in cancer immunobiology given their ability to degrade type IV collagen, a structural component of basement membranes. Degradation of this barrier facilitates tumour cell escape from the primary site. Loss of this barrier may promote tumour cell migration to distant sites. MMPs also mediate cell signalling through the release of growth factors bound to the ECM, cytokine processing, and the cleavage of cellular adhesion molecules. For instance, MMPs modulate the activation of transforming growth factor (TGF)‐β and the cleavage of E‐cadherin, potentially culminating in epithelial‐mesenchymal transitiion  (EMT), and perivascular degradation of the ECM, thereby supporting tumourigenesis. The MMP‐expressing cells may originate from tumour fibroblasts, macrophages, and tumour cells. MMPs are regulated at both transcriptional and post‐translational levels and are often induced by tumour‐associated signals, inflammation, and changes in blood flow [7, 9, 10].

Membrane‐type MMPs such as MMP14 can act directly or indirectly through the activation of other MMPs, such as pro‐MMP2, thereby inducing proteolytic cascades [11, 12]. Other than their roles in structural degradation, the uncategorized MMPs (MMP19, MMP21, MMP23, MMP27, and MMP28) represent a distinct subset of the MMP family with tissue‐specific expression patterns, although their functions remain variable and variable, relatively poorly characterised substrate specificities. For example, MMP19 and MMP21 are involved in remodelling processes during development, wound healing, and chronic hyperinflammation [13, 14]. In addition, MMP23, which lacks a conventional signal peptide and catalytic domain, may play a role in immunoregulation and neuroinflammation, possibly through the action of a cytoplasmic protein [15, 16]. MMP27 is detected mainly in immune cells and may be involved in cytokine regulation, although its precise role has not been fully elucidated. MMP28 or epilysin also appears to regulate epithelial and mesenchymal interactions and macrophage functions, and there is increasing evidence regarding its potential participation in tumour progression and repair. As new studies emerge, these lesser‐characterised MMPs are being identified as modulators of inflammation, immunity, and tumour biology, rendering them potential targets for further investigation. ECM composition and remodelling drive immune cell recruitment and phenotype in the TME. In addition, matricellular proteins and ECM stiffness influence macrophage, cancer‐associated fibroblast, and T‐cell characteristics, with effects on treatment response and tumour progression. Additionally, hypoxia inducible factor (HIF) signalling governs ECM remodelling (including collagen deposition, crosslinking, and altered matricellular expression). Therefore, ECM architecture and stiffness may influence hypoxia and HIF activation. Together, ECM remodelling, hypoxia, and immune rewiring create a self‐reinforcing niche in which these processes cooperate to concentrate proteolytic activity and further remodel the ECM in cancer [17, 18].

Although MMPs are well established as drivers of cancer progression, their functional redundancy and essential roles in normal physiology have limited the success of therapeutic targeting. Broad‐spectrum inhibitors of MMPs have performed poorly in clinical trials due to side effects arising from their lack of specificity [19, 20]. More recent strategies have focused on targeting specific MMP isoforms or their upstream regulators, including tissue inhibitors of metalloproteinases (TIMPs) [21, 22, 23], as well as ECM‐modulating proteins, such as testican‐1. Therefore, a better understanding of how testican‐1 regulates MMP activity may provide new insights into ECM remodelling in cancer. In this context, testican‐1 may function not only as a structural component but also as a scaffold or regulator influencing MMP localisation, induction, or substrate specificity. Elucidating these interactions at a molecular level could help inform precision therapeutic strategies to limit ECM degradation and metastasis.

## Materials and methods

To study testican‐1–MMP interactions, we used a set of *in silico* approaches, including molecular docking, molecular dynamics simulations (MDS) [24, 25], and system biology network [26] analysis. For various MMP–testican‐1 complexes, using zymogen forms, we evaluated binding specificity, complex stability, and broader pathway integration.

### Molecular docking

We employed the AutoDock tools for initial processing and HDock program to model the binding of testican‐1 (SPOCK1) to 28 diverse MMPs. Protein structures were obtained from the Protein Data Bank (PDB) [27] at https://www.rcsb.org/ or generated using homology‐based models. Preprocessing involved removal of water molecules and selection of flexible residues within functional regions of the testican‐1 protein. Docking scores were used to rank binding affinity using the HDock scoring function, and results were visualised using the PyMOL program [28].

### Molecular dynamics simulations

To study the stability of testican‐1–MMP complexes, MD simulations of 100 ns duration were performed using the GROningen MAchine for Chemical Simulations (GROMACS) [29, 30]. The optimal docking solution for each complex was then solvated, ionised by NPT ensemble simulation to equilibrate the system under constant particle number (N), pressure (P), and temperature (T). Simulation trajectories were analysed to quantify root mean square deviation (RMSD), root mean square fluctuation (RMSF), and H‐bonds [31]. Following energy minimisation using the steepest descent method, systems reaching an *F*_max_ < 1,000 kJ mol^−1^ nm^−1^ value were subjected to two equilibration stages under NVT (constant number of particles and temperature) and NPT (constant number of particles, presssure and temperature) constraints. Temperature was controlled using the velocity‐rescale method (310 K). For pressure coupling, we used the Parrinello–Rahman method with the pressure set to 1 bar. Long‐range electrostatic interactions were treated using the Particle Mesh Ewald (PME) method. H‐bonded constraints were enforced using the linear constraint solver (LINCS) algorithm, allowing a 2‐fs integration time step [32].

To improve robustness and reproducibility, each complex was simulated in three independent production runs with different initial velocity seeds using the same all‐atom force field parameters. The simulation parameters remained constant across all conditions. Results obtained [RMSD, RMSF, the number of H‐bonds, and molecular mechanics/generalised born surface area (MM/GBSA) energies] are presented as mean ± SEM across simulations (*n* = 3 per group).

Convergence analyses were assessed by visual inspection of RMSD profiles and trajectory block averaging analyses. Systems were considered functionally converged when RMSD values remained constant after the initial 10–20 ns without major alterations (< 0.3 nm).

RMSF and H‐bond profiles from the three independent simulations for each complex are shown in the supplementary material, Figures S5 and S6. Molecular dynamics simulations were performed using the OPLS‐AA all‐atom force field with the TIP3P water model. The systems were neutralised and supplemented with Na^+^/Cl^−^ ions to achieve a physiological concentration of 0.15 M. Following steepest‐descent energy minimisation, the systems were equilibrated under position restraints using NVT and NPT ensembles. Temperature was maintained at 310 K using the velocity‐rescale thermostat, and pressure was maintained at 1 bar using the Parrinello–Rahman barostat. Long‐range electrostatics were treated using the PME method, and bond constraints were applied using the LINCS algorithm.

For each molecular dynamics system, three independent production simulations were conducted. Additionally, RMSD, RMSF, H‐bonds, and MM/GBSA energies were reported as mean ± SEM across replicates. Convergence was assessed by RMSD plateauing and block‐averaged analyses. Finally, Define Secondary Structures of Protein (DSSP) analysis was carried out to detect secondary protein structures in each complex throughout the simulations [33].

### Systems biology modelling

We employed STRING and BioGRID databases [34, 35], to construct interaction networks involving testican‐1 and MMPs, which were visualised using Cytoscape [36]. Co‐expression and enrichment analyses were used to detect testican‐1‐related signalling pathways and regulatory molecules within the ECM. For STRING, interactions were filtered via the combined confidence score with a high‐confidence threshold (required score ≥ 0.7), and functional enrichment significance was assessed using Benjamini–Hochberg false discovery rate (FDR), with FDR < 0.05 considered statistically significant.

### Immunofluorescence staining for MMP–testican‐1 colocalisation analysis

Immunofluorescence (IF) was performed based on our previously established protocol [37]. DLD‐1 (ATCC, CCL‐221) and THP‐1 (ATCC, TIB‐202) cells cultured on glass coverslips were fixed with 4% paraformaldehyde for 15 min, permeabilised with 0.1% Triton X‐100 for 10 min, and blocked using 5% HSA in PBS for 1 h. Cells were incubated overnight at 4 °C with primary antibodies diluted in 1% BSA/PBS: SPOCK1 polyclonal antibody (Thermo Fisher Scientific, PA5‐98497) and MMP2 monoclonal antibody (mAb) (Proteintech, 66366‐1‐Ig) at 1:200. After PBS washes, specimens were incubated for 1 h at room temperature with Alexa Fluor 488‐ and Alexa Fluor 594‐conjugated secondary Ab (Thermo Fisher Scientific; 1:500) and counterstained with DAPI (1 μg ml^−1^). IF assay materials used were only supported by the University of Nottingham. Results were imaged by a Leica STELLARIS confocal fluorescence microscopy.

## Results

### Testican‐1 structural domains and functional features

**Figure 1:**
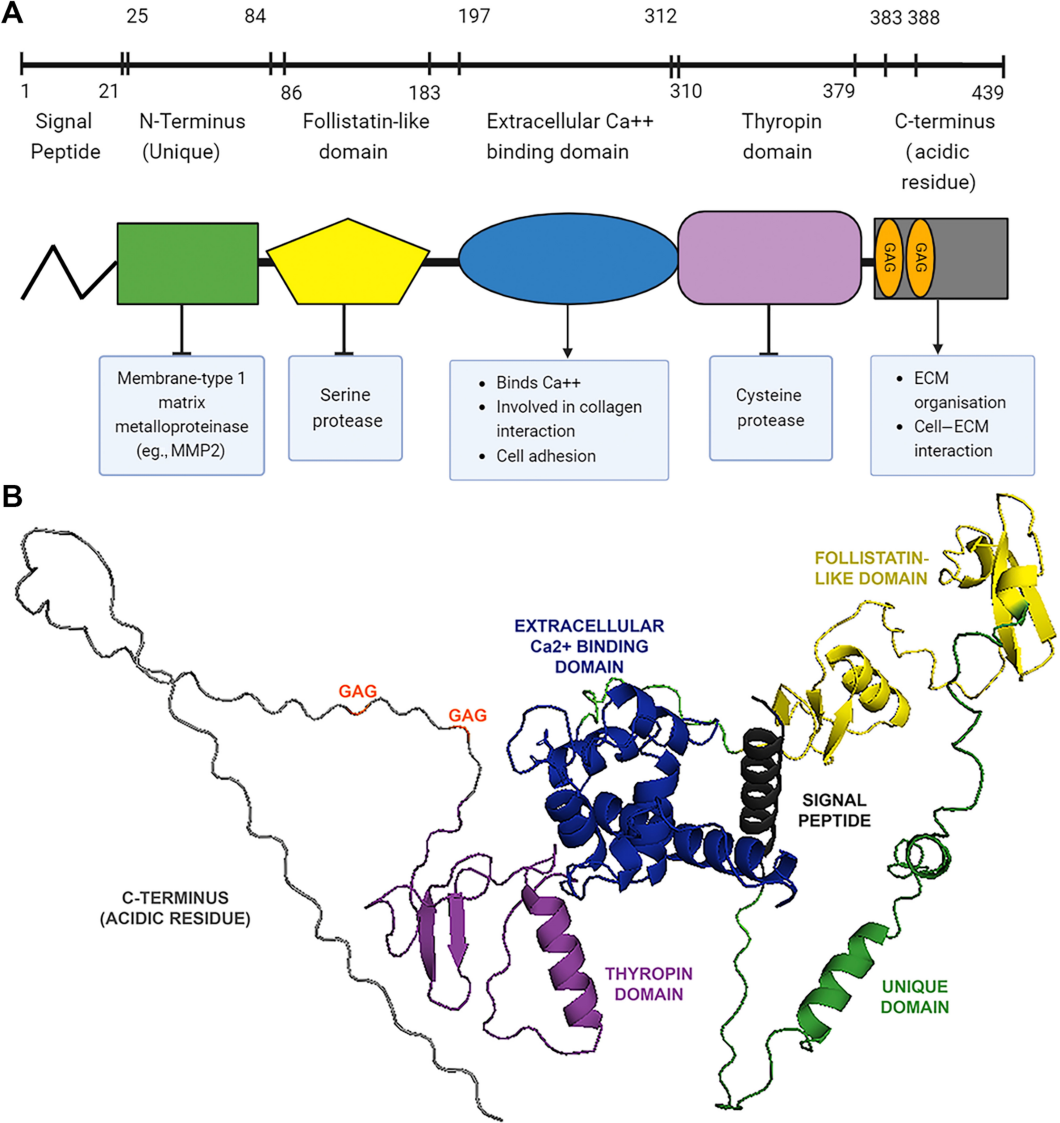
Domain organisation and predicted 3D structure of testican‐1. (A) Schematic representation of testican‐1 (SPOCK1) highlighting the major functional domains: signal peptide (residues 1–21), unique N‐terminal region, follistatin‐like domain (residues 86–183), extracellular Ca^2+^‐binding domain (residues 197–312), thyropin domain (residues 310–379), and an acidic C‐terminal domain (residues 383–439). Functional annotations indicate known or proposed interactions with MMPs, serine proteases, and roles in ECM organisation and cell–ECM interactions. Glycosaminoglycan (GAG)‐binding sites are also indicated. (B) Predicted 3D structural model of SPOCK1, with domains colour‐coded as follows: signal peptide (black), unique N‐terminal region (green), follistatin‐like domain (yellow), extracellular Ca^2+^‐binding domain (blue), thyropin domain (purple), and acidic C‐terminal region (grey). GAG‐binding sites are highlighted in red. Created with BioRender.com

Testican‐1 has multiple domains in its proteoglycan structure which participate in various biological processes. These regions comprise a secretion signal peptide, a unique N‐terminal domain, a follistatin‐like region which recognises protein–protein interactions, a Ca‐binding region which may provide structural integrity, a thyropin domain with potential protease inhibitor activity, as well as an acidic C‐terminal domain that participates in ECM interactions. The unique domain and specific motifs embedded in this protein are crucial for its ECM remodelling role since these could potentially bind proteases such as MMPs (Figure 1). Post‐translational modifications, including phosphorylation, glycosylation, ubiquitination, and acetylation, can further modulate testican‐1 functions, localisation, and degradation. Data from TCGA indicates that testican‐1 is frequently overexpressed and mutated in various cancers, including colorectal cancer, gliomas, lung cancer, and pancreatic carcinoma. Notably, mutation hotspots often occur in regions that interact with MMP proteins or in domains that are involved in ECM binding.

The regulatory complexity of testican‐1 in cancer is reflected by its diverse range of post‐translational modifications (PTMs) and the presence of various mutations (supplementary material, Figure S1A,B). SPOCK1 (testican‐1) contains multiple molecular features that are likely to influence its function within the ECM, including its localisation, stability, and interactions with key binding partners, such as MMP family proteins. The schematic representation of testican‐1 post‐translational modification, including phosphorylation, acetylation, ubiquitination, and both O‐ and N‐glycosylation. Notably, testican‐1 appears to contain multiple phosphorylation sites, especially around the thyropin domain and the acidic C‐terminal regions, suggesting that these regions may have important regulatory roles. Modifications in the unique domain may also influence testican‐1 interactions with MMP proteins. In addition, glycosylation may support proper protein folding, secretion, and subsequent incorporation into the ECM.

On comparison of the mutation frequency in various cancer types (supplementary material, Figure S1C), the frequency of testican‐1 mutations appears high in colorectal, endometrial, lung, or brain cancers, which are typically associated with severe ECM remodelling and high expression of MMPs. This high mutation prevalence suggests the functional importance of testican‐1 in these cancer types, thereby contributing to the promotion of ECM degradation and metastasis. Subsequent examination of the testican‐1 sequence indicated the presence of abundant mutation hotspots, which are found either overlapping with or immediately adjacent to regulatory PTM sites. This overlap indicates the potential influence of these mutations on PTMs, which could affect the conformational structure or protein–protein interaction features of testican‐1.

This biphasic regulatory influence at the genomic or proteomic level might have profound effects on the functional roles associated with testican‐1, including the promotion or scaffolding/modulation of MMPs. This comprehensive regulatory landscape emphasises the key effector role of testican‐1 in ECM remodelling processes. The presence of PTMs together with cancer‐associated mutations within the conserved regions indicates the functional importance of these regulatory sites. Site‐directed mutagenesis analyses in the context of malignancy models could yield significant insights into the regulatory or functional consequences associated with these modifications. Additionally, the spatial proximity between regulatory sites and mutation hotspots may provide insights into potential therapeutic targeting.

Testican‐1 may play a role in the tumour microenvironment, as contextualised by system biology network analysis implemented via STRING, BioGRID, and Cytoscape platforms (supplementary material, Figure S2A–C). The derived interaction maps place testican‐1 as a central hub within ECM remodelling interaction networks. Strong co‐expression was detected between testican‐1 and ECM proteins of interest, such as fibronectin (FN1), several collagens, and metalloproteinases (MMP2, MMP9, MMP14). Clustered co‐expression was reported with immune regulatory elements such as TGF‐β1, interleukin (IL)‐6, and STAT3, overall pointing to its involvement in immune‐suppressive tumour microenvironment. Moreover, Gene Ontology (GO) enrichment analyses emphasised biological processes such as collagen fibril arrangement, basement membrane disintegration, and cell‐matrix adhesion. Kyoto Encyclopedia of Genes and Genomes (KEGG) pathway analysis linked testican‐1 to focal adhesion, PI3K‐Akt, and TGF‐β signalling, which are important processes during cancer invasion and metastasis. These systems biology insights elucidate a multi‐faceted role for testican‐1 in linking matrix remodelling with tumour‐enhancing signalling cascades.

### Testican‐1 overexpression is associated with poor clinical outcomes

**Figure 2:**
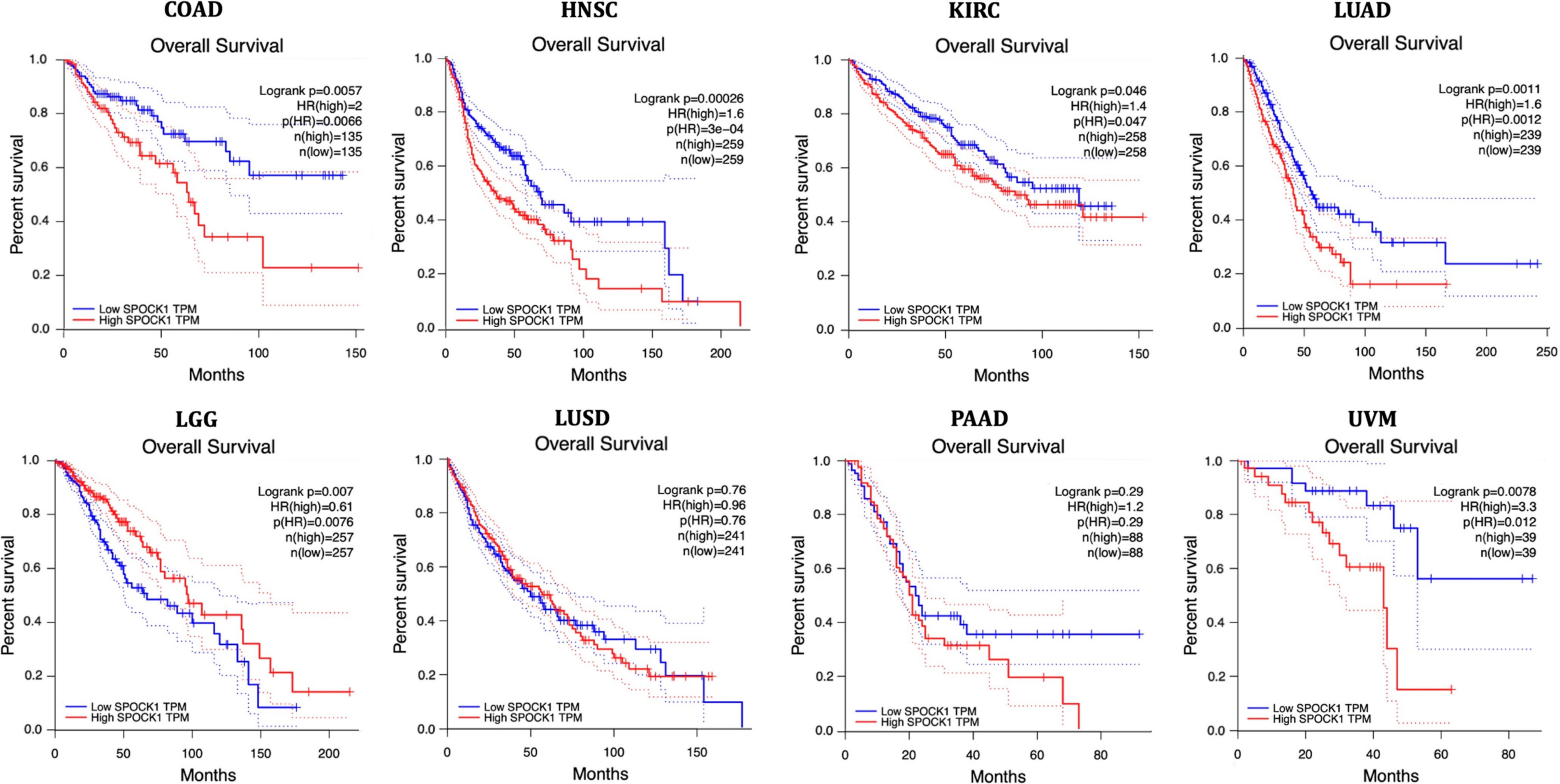
Kaplan–Meier survival analysis of testican‐1 expression in multiple cancer types. Kaplan–Meier plots showing the correlation between testican‐1 (SPOCK1) transcript expression levels and overall patient survival in eight tumour types from TCGA: COAD, HNSC, KIRC, LUAD, LGG, LUSC, PAAD, and UVM. Patients were stratified into high and low SPOCK1 expression groups based on median transcript levels. High SPOCK1 expression is associated with significantly reduced overall survival in several cancers. *p* values were calculated using the log‐rank test. Data were visualised via GEPIA2. HNSC: *n* = 518 (*n* ^high^ = 259, *n* ^low^ = 259); HR^high^ = 1.6; log‐rank *p* = 0.00026; *p*(HR) = 3e−04. KIRC: *n* = 516 (*n* ^high^ = 258, *n* ^low^ = 258); HR^high^ = 1.4; log‐rank *p* = 0.046; *p*(HR) = 0.047. LUAD: *n* = 478 (*n* ^high^ = 239, *n* ^low^ = 239); HR^high^ = 1.6; log‐rank *p* = 0.0011; *p*(HR) = 0.0012. LGG: *n* = 514 (*n* ^high^ = 257, *n* ^low^ = 257); HR^High^ = 0.61; log‐rank *p* = 0.007; *p*(HR) = 0.0076. LUSC: *n* = 482 (*n* ^high^ = 241, *n* ^low^ = 241); HR^high^ = 0.96; log‐rank *p* = 0.76; *p*(HR) = 0.76. PAAD: *n* = 176 (*n* ^high^ = 88, *n* ^low^ = 88); HR^high^ = 1.2; log‐rank *p* = 0.29; *p*(HR) = 0.29. UVM: *n* = 78 (*n* ^high^ = 39, *n* ^low^ = 39); HR^high^ = 3.3; log‐rank *p* = 0.0078; *p*(HR) = 0.012.

GEPIA2 was selected to plot Kaplan–Meier survival graphs for several malignancy types, including colorectal [colorectal adenocarcinoma (COAD)], head and neck [head and neck squamous carcinoma (HNSC)], renal [kidney renal cell carcinoma (KIRC)], lung [lung adenocarcinoma (LUAD)], LUAD and lung squamous cell carcinoma (LUSC), low‐grade glioma (LGG), pancreatic adenocarcinoma (PAAD), and uveal melanoma (UVM). Increased testican‐1 expression predicted worse survival, with statistically significant separation of high‐ and low‐expression cohorts (Figure 2). These data suggest testican‐1 may serve as a plausible predictive marker.

### Molecular docking and dynamics of testican‐1–MMP complexes

Optimal testican‐1–MMP interactions were examined using the HDock docking tool. The crystal configurations of MMP isoforms were obtained from the PDB, and a homology model was generated for testican‐1; alternatively, the models were predicted using the Alphafold server, which has been used in studies to model unknown protein structures and assess the effects of sequence modifications [38, 39, 40]. Docking results indicated that the unique domain and the C‐terminal acid portion of testican‐1 often bind to the catalytic and hemopexin domains of MMPs (supplementary material, Figure S3). These testican‐1–MMP interactions suggest that testican‐1 may function as a regulatory factor influencing MMP substrate accessibility or enzyme activity.

The testican‐1‐specific domain was docked *in silico* to evaluate optimal binding poses with members of the MMP family using the HDOCK server. Protein–protein docking results are summarised in the supplementary material, Table S2, and show a range of stable docking scores for testican‐1 across MMP members (−343.38 to −214.97), with lower docking scores indicating more stable binding. Relatively weaker affinity interactions were found with the classical collagenases and gelatinases MMP2, MMP3, and MMP13, suggesting a more selective binding interface between testican‐1 and either the membrane type or secreted subgroups of MMPs.

Among the 28 MMPs investigated, the strongest predicted interactions with testican‐1 were observed for MMP23, MMP25, and MMP28, suggesting that these interactions may have greater biological significance. These MMPs have been implicated in processes such as immunity, wound healing, and tumour invasion, consistent with reported roles of testican‐1 in ECM remodelling and cancer.

Collectively, these findings support the hypothesis that testican‐1 may interact with or modulate the activity of a subset of MMPs in the tumour microenvironment, particularly those functioning in pericellular proteolysis and inflammatory remodelling. Details of MMP zymogen sequences, curated against Uniprot (https://www.uniprot.org/) and HGNC (HUGO; https://www.genenames.org/), are provided in the supplementary material, Table S3. These data provide a framework for prioritising MMP–testican‐1 complexes for further experimental validation and therapeutic investigation.

**Figure 3:**
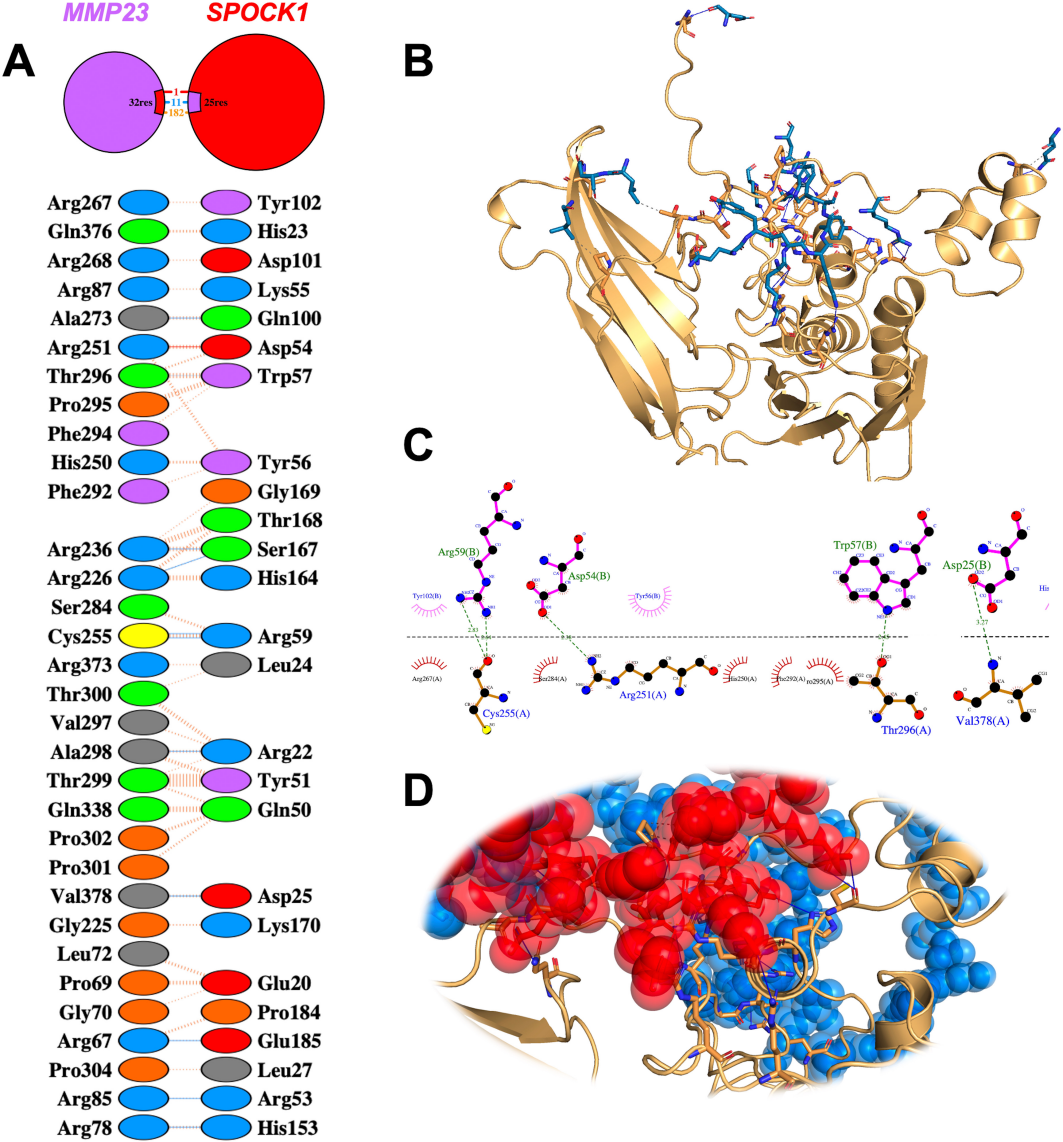
Structural and molecular interaction analysis of MMP23–testican‐1 binding interface. (A) Schematic diagram showing predicted residue‐level interactions between MMP23 (purple) and testican‐1 (SPOCK1) (red). Amino acids from each protein involved in the interaction are colour‐coded and connected by dashed lines. (B) Three‐dimensional ribbon representation of MMP23–testican‐1 complex, with MMP23 shown in gold and key interacting residues highlighted in stick representation. (C) Two‐dimensional interaction plots depicting representative hydrogen bonds and polar interactions stabilising the interface. Key residue pairs such as Arg98(B)–Asp48(B), Trp57(B)–Arg251(A), and Asp25(B)–Val378(A) are shown with interaction distances. (D) Surface rendering of protein–protein interaction interface, with MMP23 residues in blue and testican‐1 residues in red, highlighting the dense and complementary surface involved in binding.

Interacting residues within the testican‐1 unique domain (residues 25–84) and the three top‐scoring MMPs (MMP23, MMP25, and MMP28) were plotted using LigPlot+ (version 2.2) [41, 42]. Hydrogen bonds, non‐bonded interactions, disulphide bonds, and salt bridges were quantified using the PDBSum tool with default settings [4, 43, 44]. To further characterise the molecular basis of the MMP23–SPOCK1 interaction, structural docking analysis revealed a well‐defined binding interface comprising several stabilising interactions (Figure 3). In this complex, 32 residues of MMP23 were predicted to interact with 35 residues of testican‐1. Surface mapping of the complex identified key residues on MMP23, including Arg251, Thr296, and Cys255, interacting with important sites on testican‐1 such as Trp57, Asp48, and Asp25. These interactions occurred in the extracellular region of the testican‐1 protein, which is in line with the secretory role of testican‐1. However, further investigation using active MMP forms is warranted to validate these findings.

Three‐dimensional modelling analysis showed that MMP23 retains its core catalytic domain structure and binds testican‐1 within a complementary grooved surface; however, further validation using different activation states of MMPs is warranted. Two‐dimensional interaction plots emphasised the predominance of polar interactions which may contribute to specificity and affinity. The surface plots of the interfaces showed a high degree of binding complementarity, suggesting that testican‐1 may interact with MMP23 to modulate its activity and facilitate coordinated functions in extracellular matrix remodelling.

Overall, these results suggest that the MMP23–testican‐1 complex may represent a potential target for modulating tumour microenvironment signalling and may contribute to therapeutic intervention in colorectal cancer and other epithelial malignancies.

**Figure 4:**
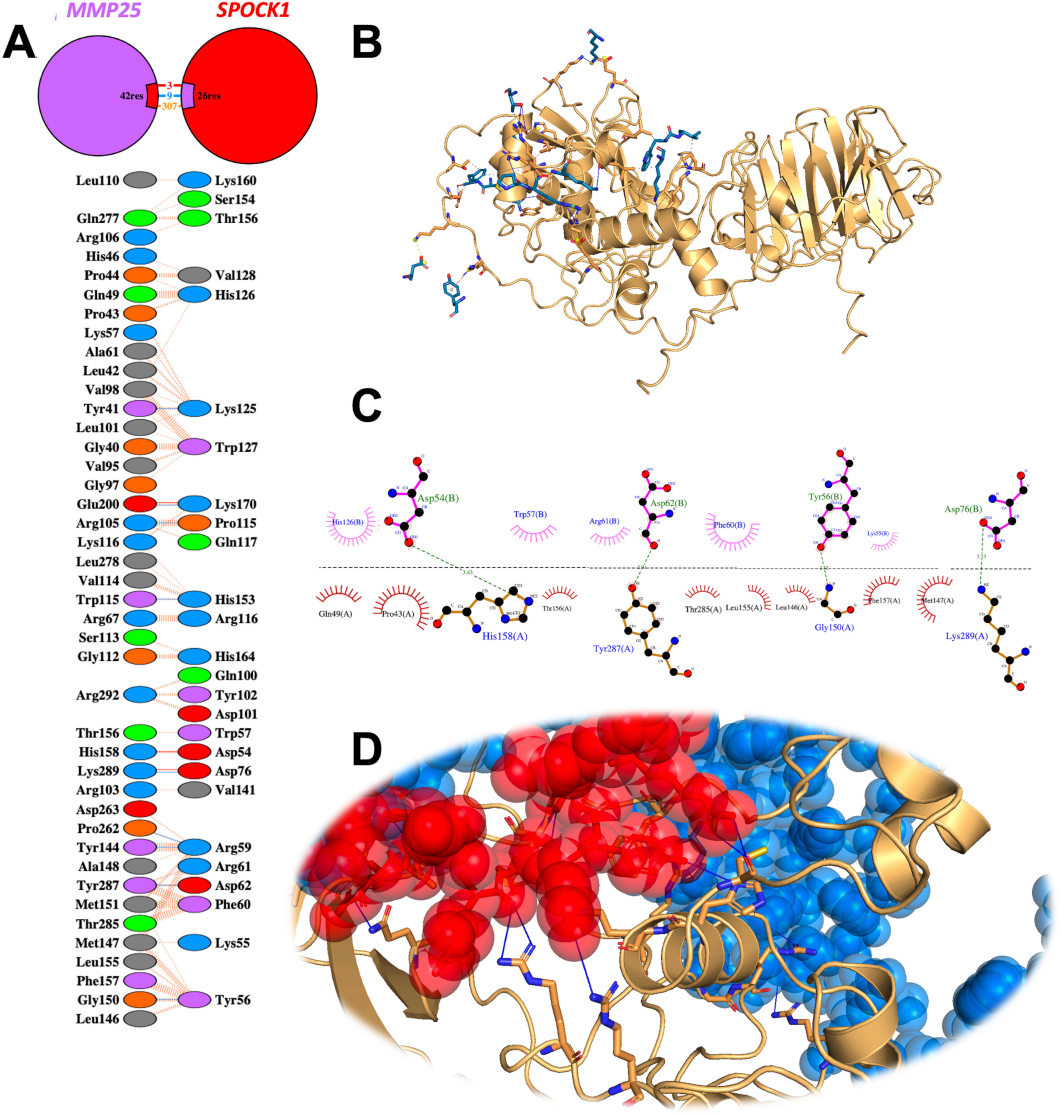
Molecular interaction analysis between MMP25 and testican‐1 (SPOCK1). (A) Schematic on left illustrates predicted residue‐level interactions between MMP25 (purple) and testican‐1 (SPOCK1) (red) based on docking simulations. Interacting amino acids are represented by coloured ovals and connected by dashed lines, with MMP25 residues listed on the left and SPOCK1 residues on the right. (B) Overall structure of predicted MMP25–SPOCK1 complex, with MMP25 rendered as a ribbon model (gold) and interacting side chains in stick representation (blue). (C) Detailed 2D interaction maps of selected residue pairs, highlighting hydrogen bonds and interaction distances. Notably, interactions involving Asp94(B), Tyr87(A), His158(A), and Lys259(A) are shown to stabilise the interface. (D) Surface rendering of binding pocket, with interacting regions of SPOCK1 and MMP25, illustrating compactness and complementarity of binding site.

To investigate the structural binding interface of the MMP25–testican‐1 complex, we employed molecular docking and binding interface mapping analyses (Figure 4). This revealed a rather large binding interface, with over 30 binding residues for both proteins. This binding interface was characterised by a network of H‐bonds and hydrophobic interactions, with contribution from residues Asp94(B), Tyr87(A), and His158(A) from MMP25 and Gln100, Asp101, and Trp127 from testican‐1. Detailed analysis of the binding interface also identified conserved motifs within the ECM domains of testican‐1, which may regulate protease activity and tumour microenvironment remodelling.

**Figure 5:**
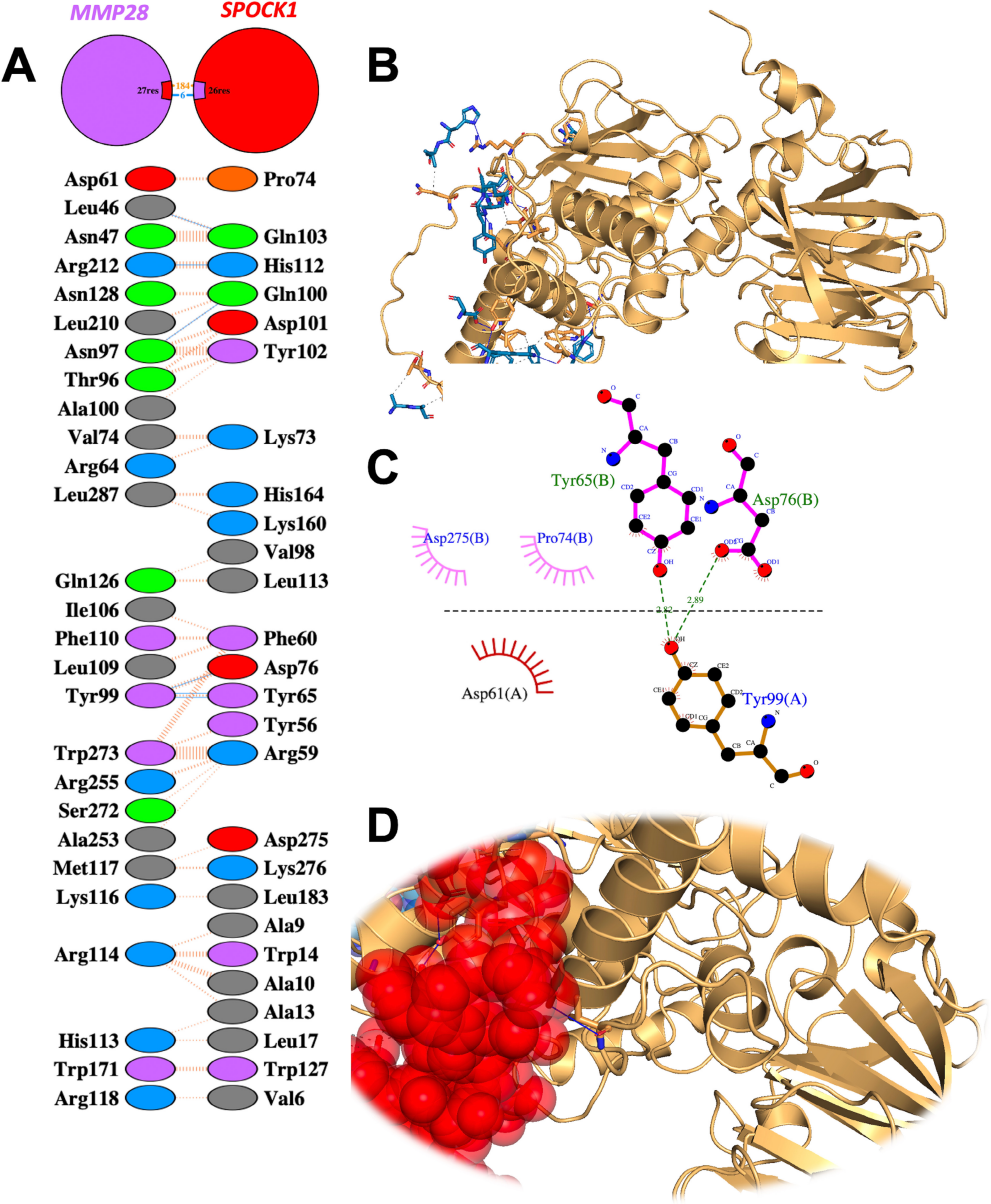
Predicted interaction interface between MMP28 and testican‐1 (SPOCK1) proteins. (A) Schematic illustrating predicted residue‐level interactions between MMP28 (purple) and testican‐1 (SPOCK1) (red) based on molecular docking analysis. Each interacting residue pair is indicated by lines, with amino acids from MMP28 shown on left and those from SPOCK1 on right. (B) Overall predicted complex structure of MMP28 (gold ribbon) bound to SPOCK1, with interacting residues visualised in stick representation. (C) This panel illustrates key hydrogen bond interactions between specific residues, including Asp76(B)–Tyr65(B) and Tyr99(A)–Asp76(B), highlighting distances in angstroms. (D) Magnified surface representation of binding interface, with interacting residues to emphasise the core binding site.

To investigate the molecular interactions between MMP28 and testican‐1, molecular docking was performed, revealing a defined binding interface characterised by H‐bonds and hydrophobic interactions (Figure 5). The key binding residues identified included Asp76, Tyr65, and Tyr99 in testican‐1, which form favorable binding interactions with corresponding residues in MMP28. The model suggests that MMP28 engages it conventional catalytic domain, while testican‐1 uses its secretory domains, indicating a potential functional interaction that could alter extracellular matrix remodelling.

The binding residues identified by the structural models and binding schematics may provide potential sites for therapeutic intervention. Moreover, this binding interface appears to contain closely packed hydrophobic sites, further supporting the specificity of the MMP28–testican‐1 interaction. Collectively, these testican‐1–MMPs interactions may drive tumour ECM remodelling and immune evasion (supplementary material, Figure S4).

### Molecular dynamics simulation analysis confirms stable binding within MMP–testican‐1 complexes

The dynamics of testican‐1 interactions with MMP2, MMP25, and MMP28 were evaluated. For all simulations, RMSD convergence was achieved (supplementary material, Figure S5A). RMSF fluctuation analysis showed relative stability of the MMP unique domain region within the testican‐1 protein, which may correlate with stronger and more persistent binding to MMP molecules (supplementary material, Figure S5B–E). DSSP analysis revealed that the secondary structures of all three MMP–testican‐1 complexes were maintained throughout the simulations, with comparable distributions of α‐helices, β‐strands, and loop regions. Minor differences in helical and β‐structural elements among the complexes likely reflect adaptive conformational rearrangements upon binding (supplementary material, Figure S6A–C), consistent with observed bonding and energetic measures.

**Figure 6:**
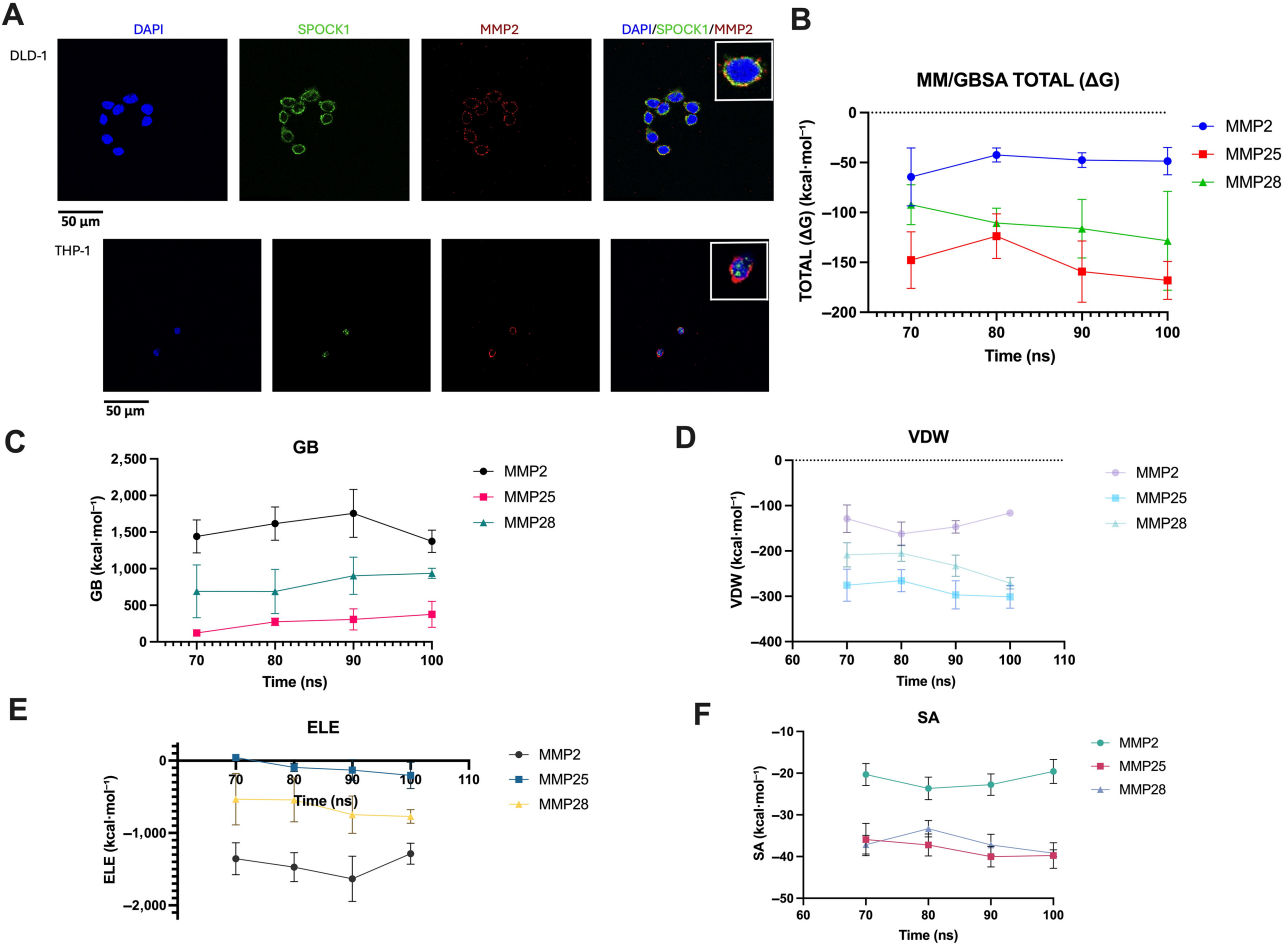
Binding‐free energy and colocalisation immunofluorescence assay in cancer cells for testican‐1–MMP2 complex. (A) Immunofluorescence of DLD‐1 and THP‐1 cells stained with DAPI, testican‐1 (SPOCK1), and MMP2, with merged channels (top‐right panels, maginified cell images) (B) MM/GBSA total binding free energy (ΔG) for MMP2, MMP25, and MMP28 over time. (C) Generalised Born (GB) solvation energy. (D) Van der Waals (VDW) interaction energy. (E) Electrostatic (ELE) interaction energy. (F) Solvent‐accessible surface area (SA) contribution.

Equilibrated frames of the dynamic simulation trajectories indicated that all three complexes formed stable interactions between MMP and testican‐1, with H‐bonds preserved throughout the dynamics. MMP2, MMP25, and MMP28 exhibited mean bond numbers of 17.76 ± 0.27, 22.22 ± 1.28, and 24.35 ± 3.48, respectively, indicating robust and sustained binding across the complexes (supplementary material, Figure S6D–G). Additionally, MM/GBSA analyses showed favourable negative binding energies across all stabilised frames, with mean binding energies of −50.71 ± 4.75, −149.7 ± 9.61, and − 111.9 ± 7.53 kcal mol^−1^ for MMP23, MMP25, and MMP28, respectively, indicating energetically stable complex formation. Finally, experimental validation by colocalisation assays provided additional confirmation of these predicted interactions (Figure 6).

### Immunofluorescence assay suggests MMP–testican‐1 colocalisation

Colocalisation of MMP2 and testican‐1 was performed to provide further validation for interaction of MMPs and their involvement in remodelling in cancer. DLD‐1 human colorectal epithelial cancer and THP‐1 human monocytic leukemia cell lines were used to examine MMP2 and testican‐1 expression patterns. Results showed that testican‐1 (green) and MMP2 (red) colocalised in DLD‐1 CRC cells but not in THP‐1 monocytes (Figure 6A), consistent with previous observations suggesting that THP‐1 monocytes do not synthesise their own ECM [45]. Together with molecular docking and dynamic simulation data (Figure 6B–F), these findings suggest that testican‐1 may modulate ECM remodelling through interactions with MMPs, however, further experimental validation is required.

## Discussion

The ECM is increasingly recognised as an active participant in cancer progression due to its ability to shape the biochemical and mechanical processes which influence tumour invasion, metastasis, and immune escape. Using structural modelling, dynamic simulation, and systems biology approaches, our study provides mechanistic insights into the role of testican‐1 in regulating MMPs activity within the tumour microenvironment. We used molecular docking, MDS, and systems biology network interactions analyses to suggest that testican‐1 could potentially interact with MMP23, MMP25, and MMP28 – enzymes increasingly implicated in the processes of ECM remodelling, immunomodulation, and tumour dissemination.

Cancers are influenced by dysregulation in various immune system elements [46, 47]. Earlier research revealed that testican‐1 expression levels were higher in a broad spectrum of human cancers, in which it exerted a role in enhancing EMT, migration, and matrix destruction [2, 3, 48, 49]. For instance, Lin *et al* demonstrated that testican‐1 increased MMP2 activity in renal cell carcinoma cells via a Snail/Snag axis to trigger EMT and invasiveness in cells [2]. Moreover, Chien *et al* reported that suppression of testican‐1‐regulated pathways inhibited the metastatic potential in prostate carcinoma cells [3]. Although previous studies indicated regulatory interactions between testican‐1 and MMPs, in this work, we establish for the first time a structural basis for demonstrating that testican‐1 can bind to and stabilise MMPs, with greater affinities observed for MMP23, MMP25, and MMP28.

MMPs classified as ‘uncharacterised and atypical MMPs’ are less well investigated in comparison with gelatinases MMP2 and MMP9. Nevertheless, recent findings suggest that they play important roles in pericellular proteolysis, immune regulation, and ECM remodelling. Indeed, MMP23 is believed to lack a typical secretory signal peptide, and studies have shown that it participates in the regulation of immune cells and cytokine maturation [50]. There is also evidence that MMP28 plays a role in macrophage polarisation and tissue repair, in addition to showing elevated expression in various cancers [51].

Molecular docking analysis in this study suggests strong binding between MMPs and both the unique domain and acidic domain of testican‐1, further supporting its role as a protein scaffold in ECM regulation. Moreover, molecular dynamics studies were carried out to further examine and validate the stability of the docked complexes. This was demonstrated by the presence of considerable H‐bonds throughout the molecular dynamics simulation runs.

Overall, these findings lend support to the hypothesis that testican‐1 binds to MMPs and may facilitate MMP localisation, accessibility, or stability within the ECM. Importantly, the identified binding interfaces contain mutation hotspots associated with human cancer, further suggesting that tumoural pathologies may be affected by dysregulation of ECM remodelling in these regions.

STRING and BioGRID network analysis indicates interactions involving testican‐1 in the context of ECM degradation and immunoregulation. Testican‐1 is predicted to cluster with collagens, integrins, MMPs, and immunosuppressive factors such as IL‐6, STAT3, and TGF‐β1, which are characteristic of chronic inflammation and may promote tumour progression [3, 48, 49, 52, 53, 54, 55, 56].

These findings are consistent with previous studies showing that high expression of testican‐1 is a poor prognostic factor in various cancers, including colorectal, glioma, and pancreatic cancers. Kaplan–Meier analysis derived from TCGA further support the potential clinical relevance of testican‐1. However, further independent cohort validation in cancers is warranted.

Despite substantial evidence supporting the idea that MMPs are involved in the progression of malignancies, the application of MMP inhibitors in the clinic has been limited due to issues of non‐specificity and off‐target effects. Our data suggest that targeting the interaction between testican‐1 and MMPs may provide a more specific therapeutic strategy rather than directly targeting the enzymatic activity of MMPs. The identification of novel binding pockets within the testican‐1 protein, particularly within the unique and acidic domains, highlights potential regions that could be used to specifically target the protein using small molecules or biological therapeutics. Previous studies also indicated that miRNA inhibition (anti‐miR‐135) might reduce testican‐1 function and restore drug sensitivity in colorectal malignancies [52].

Additional validation of functional interactions in patient‐derived tumour organoids, xenograft models, and genetically modified mouse models is warranted to establish the biological relevance of testican‐1 and MMP interactions identified by this analysis. Further research may include spatial analysis of transcriptomics and proteomics to better define the roles of testican‐1 within specific tumour microenvironments.

The roles of PTMs and cancer‐related mutations localised to the functional domains of testican‐1 also require investigation in the context of MMP binding and ECM regulation. Finally, this work establishes a computational framework highlighting the potential interactions of testican‐1 with MMPs in ECM remodelling. This may open new avenues for future therapeutic research in aggressive, ECM‐rich tumours, such as those observed in colon, lung, and pancreatic cancers.

## Author contributions statement

SY, KS and ASN were responsible for study conception and design. SY, KS, PK and AM developed the methodology. SY, KS, PK and AM acquired the data. SY, KS, PK, AM, LVD and ASN analysed and interpreted the data. SY, KS and ASN were responsible for writing and/or revision of the manuscript. SY, KS and ASN provided administrative, technical or material support. ASN supervised the study. All authors have read and agreed to the published version of the manuscript.

## Supporting information


**Text S1.** Introductory information on role of ECM and MMPs in cancer
**Figure S1**. Post‐translational modification sites and mutational landscape of testican‐1 (SPOCK1) protein across human cancers
**Figure S2**. Network‐based analysis of testican‐1 (SPOCK1) interaction partners and functional associations
**Figure S3**. Molecular docking analysis of testican‐1 (SPOCK1) with various MMPs
**Figure S4**. SPOCK1–MMP interactions drive tumour ECM remodelling and immune evasion
**Figure S5**. Regional flexiblity and evolution of SPOCK1–MMP2/MMP25/MMP28 during molecular dynamics simulations
**Figure S6**. H‐bond and secondary structural state of SPOCK1–MMP2/MMP25/MMP28 during molecular dynamics simulations
**Table S1**. Classification of MMPs by structure and substrate specificity
**Table S2**. Binding scores of MMP family proteins with testican‐1 (SPOCK1) unique domain
**Table S3**. Summary of pro‐MMP forms audited against Uniprot and HUGO

## Data Availability

The raw data supporting the conclusions of this article will be made available by the authors only on reasonable request.
