# Supplementary material for: Testican‐1‐MMP axis in tumour extracellular matrix (ECM) remodelling: interaction dynamics analysis and an in silico perspective
Source: J Pathol. 2026 May 15;269(4-5):433–45. doi: 10.1002/path.70073 (PMC13341274; doi:10.1002/path.70073)
Supplement: Supplementary file 1 — Text S1. Introductory information on role of ECM and MMPs in cancer Figure S1. Post‐translational modification sites and mutational landscape of testican‐1 (SPOCK1) protein across human cancers Figure S2. Network‐based analysis of testican‐1 (SPOCK1) interaction partners and functional associations Figure S3. Molecular docking analysis of testican‐1 (SPOCK1) with various MMPs Figure S4. SPOCK1–MMP interactions drive tumour ECM remodelling and immune evasion Figure S5. Regional flexiblity and evolution of SPOCK1–MMP2/MMP25/MMP28 during molecular dynamics simulations Figure S6. H‐bond and secondary structural state of SPOCK1–MMP2/MMP25/MMP28 during molecular dynamics simulations Table S1. Classification of MMPs by structure and substrate specificity Table S2. Binding scores of MMP family proteins with testican‐1 (SPOCK1) unique domain Table S3. Summary of pro‐MMP forms audited against Uniprot and HUGO [file PATH-269-433-s001.docx]

Testican-1–MMP axis in tumour extracellular matrix (ECM) remodelling: interaction dynamics analysis and an *in silico* perspective

S Yousseﬁ, K Saleki, *et al. J Pathol* <https://doi.org/10.1002/path.70073>

Supplementary Text

Supplementary Figures S1–S6

Supplementary Tables S1–S3

Reference numbers refer to the main text

**Supplementary Text**

**Introductory information on the role of ECM and MMPs in cancer**

The ECM constitutes a complicated and dynamic network of proteins, glycoproteins, and proteoglycans that functions as structural and biochemical support to surrounding cells. Besides serving as a physical scaﬀold, the ECM plays a pivotal role in regulating cell behaviours, including proliferation, diﬀerentiation, and migration. In the context of cancer, ECM dynamics are pivotal in modulating tumour cell behaviour, particularly in processes such as metastasis, invasion, and angiogenesis [1–3]. The structural components of the ECM can either inhibit or promote tumour progression, depending on their composition, organisation, and interaction with cellular receptors [1,4,5].

Metastasis, the dissemination of cancer cells from a primary tumour to distant sites, is highly inﬂuenced by ECM remodelling, which provides a permissive environment for cancer cells to detach, invade, and establish secondary tumours [2,4,6,7]. The ECM also inﬂuences invasion, where cancer cells penetrate surrounding tissues; the degradation of ECM components by enzymes such as matrix metalloproteinases (MMPs) facilitates this invasive process. Additionally, the ECM plays a crucial role in angiogenesis, the formation of new blood vessels, which supplies tumours with nutrients and oxygen essential for growth [3,8,9]. Understanding ECM dynamics and its regulatory mechanisms is thus essential for elucidating the pathways that contribute to tumour aggressiveness and identifying potential therapeutic targets in cancer treatment [3,7,9–11].


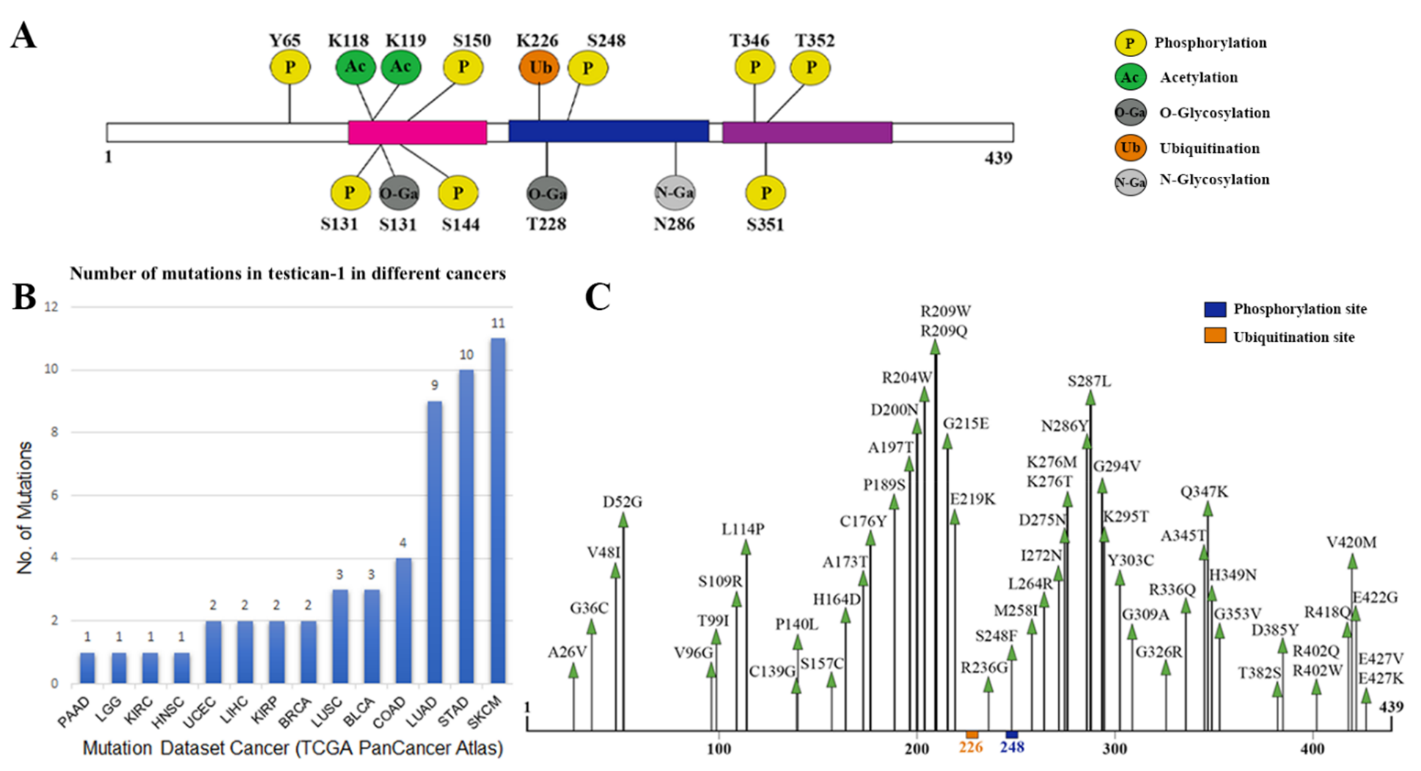


**Figure S1. Post-translational modiﬁcation sites and mutational landscape of testican-1 (SPOCK1) protein across human cancers.** (A) Schematic representation of known and predicted post-translational modiﬁcations in SPOCK1, including phosphorylation (yellow circles), acetylation (green), O-glycosylation (grey), N-glycosylation (silver), and ubiquitination (orange). Annotated sites are mapped onto key functional domains of the protein. (B) Bar graph illustrates the frequency of SPOCK1 mutations across various cancer types based on data from TCGA’s PanCancer Atlas. The highest mutation rates are observed in SKCM, STAD, and LIHC cohorts. (C) Distribution of point mutations along the SPOCK1 protein sequence, highlighting recurrent mutation hotspots. Known phosphorylation and ubiquitination sites are also marked, indicating potential regions of functional signiﬁcance.

**
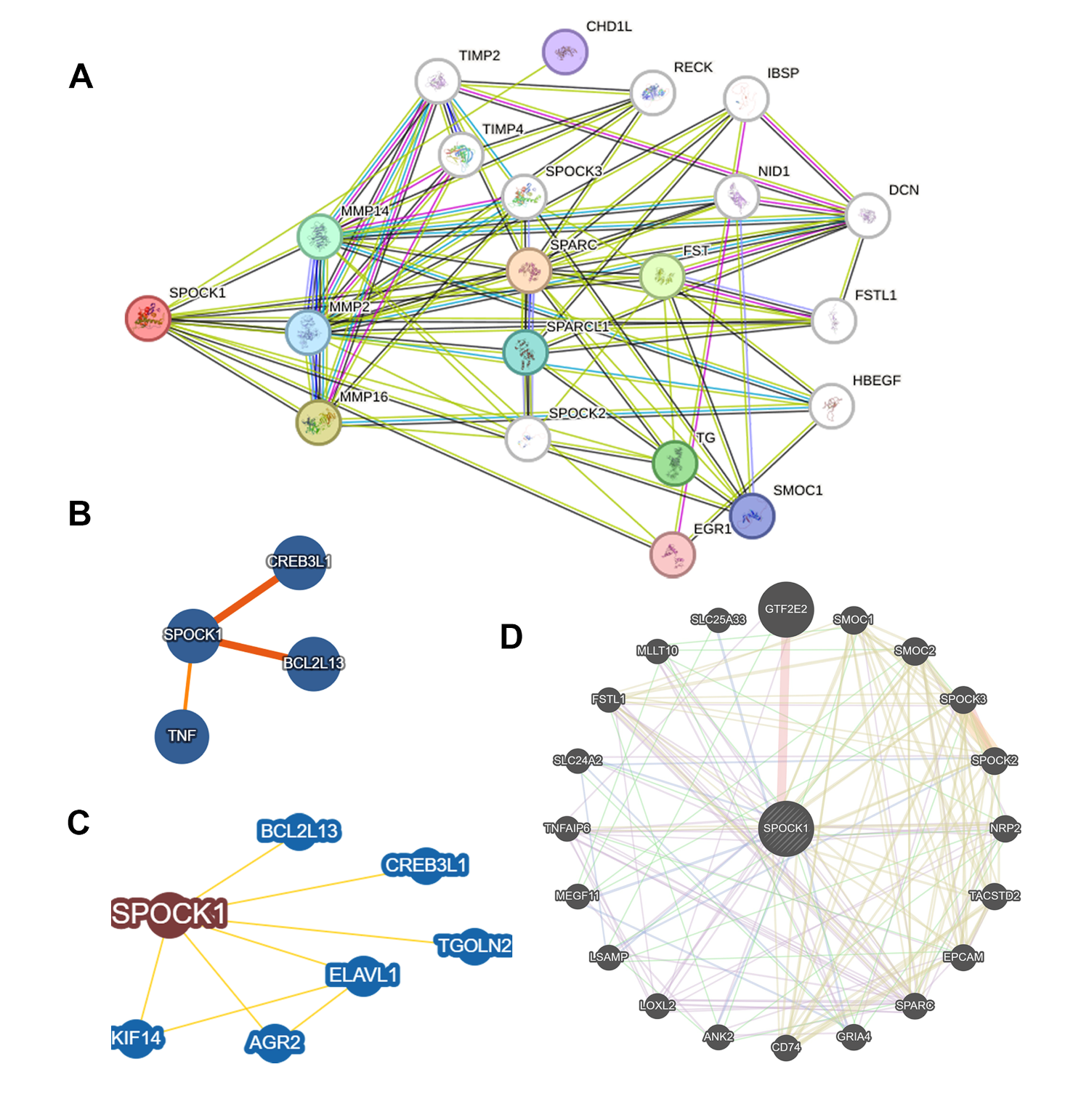
**

**Figure S2. Network-based analysis of testican-1 (SPOCK1) interaction partners and functional associations.** (A) Protein--protein interaction network showing testican-1 (SPOCK1) connectivity with ECM-related components and metalloproteinases, generated using STRING database. (B) Functional enrichment analysis highlighting predicted interaction of testican-1 (SPOCK1) with immune-regulatory proteins. (C) Gene co-expression network identifying transcriptional partners of SPOCK1. (D) Broader systems-level integration depicting SPOCK family protein interconnectivity and associated gene clusters.

**
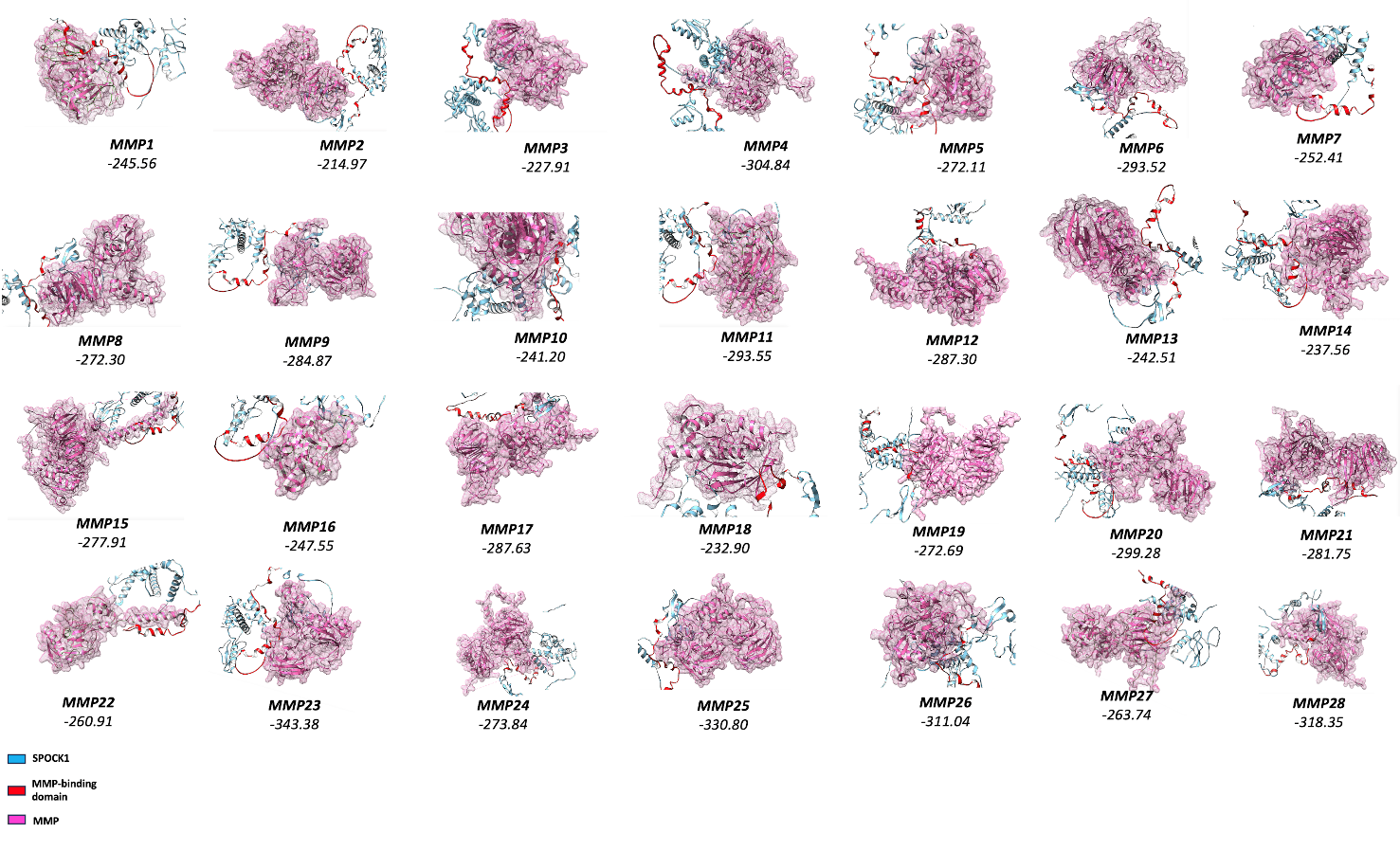
**

**Figure S3. Molecular docking analysis of testican-1 with various MMPs.** Representative docking models of testican-1 (SPOCK1) with 28 MMPs are shown. SPOCK1 is depicted in cyan cartoon representation, MMPs in pink surface representation, and predicted interaction interfaces highlighted in red. Docking scores are displayed beneath each complex, with the most favourable interactions observed for MMP23 (−343.38), MMP25 (−330.80), and MMP28 (−318.35), indicating strong predicted binding aﬃnities. These results support a potential regulatory role for SPOCK1 in modulating MMP activity during ECM remodelling.


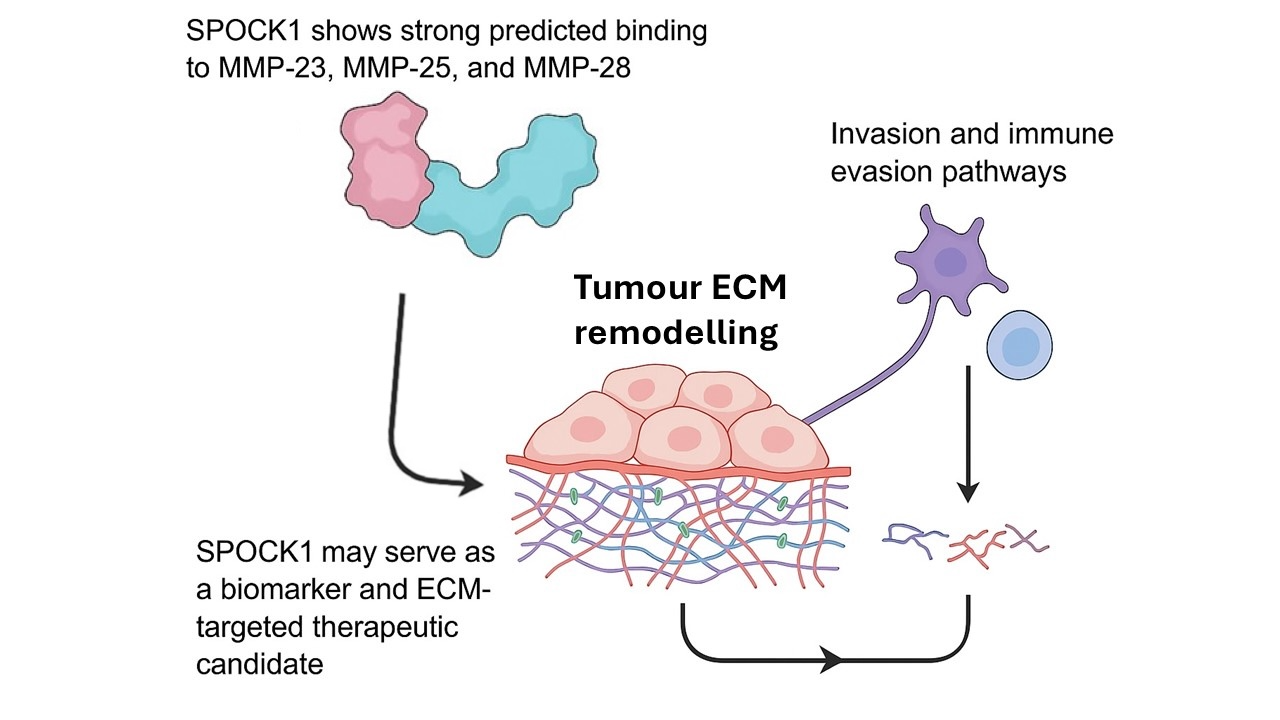


**Figure S4. SPOCK1–MMP interactions drive tumour ECM remodelling and immune evasion.** Schematic summary showing predicted interactions of testican-1 (SPOCK1) with MMP-23, MMP-25, and MMP-28, supporting a role in tumour ECM remodelling. These interactions may promote invasion, alter immune-related pathways, and highlight testican-1 (SPOCK1) as a potential biomarker and ECM-targeted therapeutic candidate. Created with BioRender.com; Created in BioRender. Saleki, K. (2026) <https://BioRender.com/d4v6jnz>


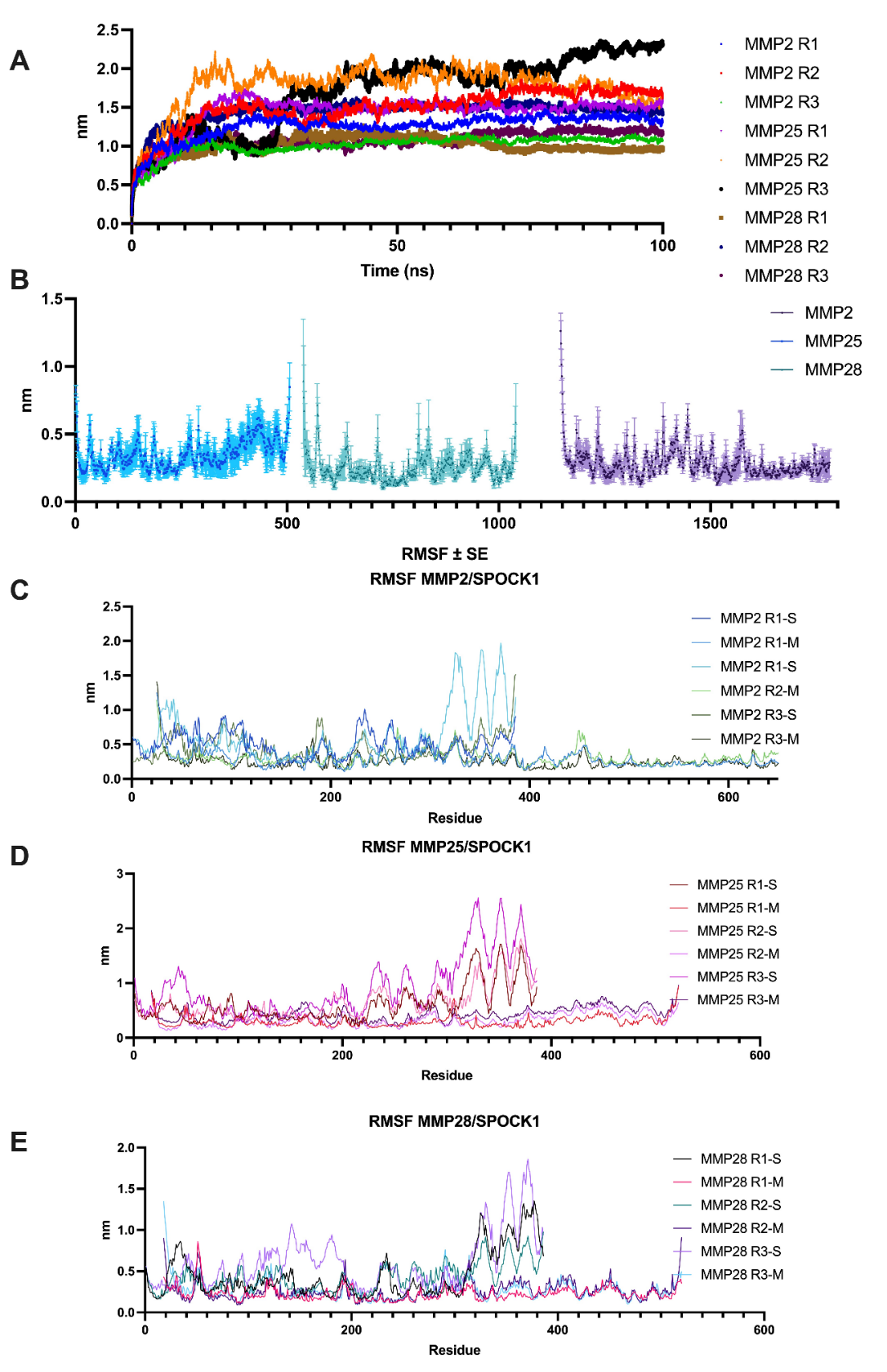


**Figure S5.** **Regional flexibility and evolution of SPOCK1–MMP2/MMP25/MMP28 during molecular dynamics simulations.** (A) RMSD of all molecular dynamics systems. (B) RMSF analysis of MMP2, MMP25, and MMP28. (C) RMSF analysis of MMP2–SPOCK1, (D) MMP25–SPOCK1, and (E) MMP28–SPOCK1 complexes.


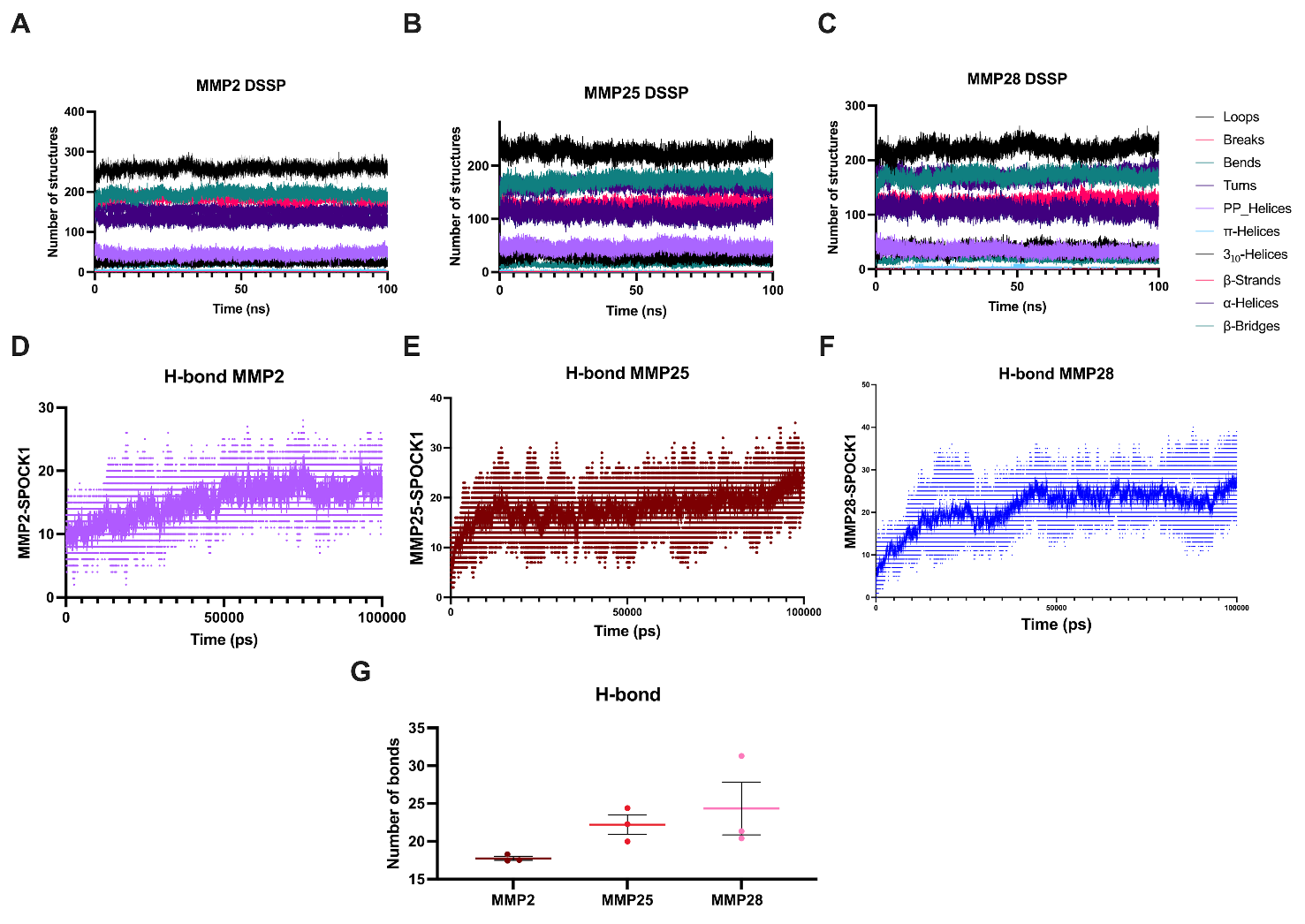
**Figure S6.** **H-bond and secondary structural state of SPOCK1–MMP2/MMP25/MMP28 during molecular dynamics simulations.** Secondary structure analysis of (A) MMP2, (B) MMP25, and (C) MMP28; H-bond numbers throughout the trajectory for testican-1 (SPOCK1) interaction with (D) MMP2, (E) MMP25, and (F) MMP28. (G) Number of H-bonds in molecular dynamics equilibrated frames of SPOCK1–MMP complexes. Data presented as mean (SEM).

### Table S1. Classiﬁcation of MMPs by structure and substrate speciﬁcity.

| **Group** | MMPs | Primary substrates | Key functions |
| --- | --- | --- | --- |
| Collagenases | MMP1, MMP8, MMP13 | Interstitial collagens (I, II, III) | Degradation of fibrillar collagen |
| Enamelysin | MMP20 | Amelogenin, enamel matrix proteins | Tooth development |
| Gelatinases | MMP2, MMP9 | Gelatin, collagen IV, elastin, fibronectin | Basement membrane remodelling, angiogenesis |
| Matrilysins | MMP7, MMP26 | Gelatin, laminin, fibronectin, proteoglycans | Wound healing, mucosal defence |
| Membrane-type MMPs (MT-MMPs) | MMP14 (MT1), MMP15–17, MMP24, MMP25 | Collagen, gelatin, laminin, ECM-bound proteins | Pericellular proteolysis, cell migration, invasion |
| Metalloelastase | MMP12 | Elastin, fibronectin, laminin, gelatin | Macrophage-mediated ECM remodelling |
| Stromelysins | MMP3, MMP10, MMP11 | Proteoglycans, fibronectin, laminin, elastin, gelatin | Broad ECM degradation; activation of other MMPs |
| Unclassified/other | MMP19, MMP21, MMP23, MMP27, MMP28 | Variable, less defined substrates | Tissue-specific or emerging roles in inflammation, immunity, cancer |

**Table S2.** Binding scores of MMP family proteins with testican-1 (SPOCK1) unique domain.

| **MMP** | **Brief description of function** | **Binding**  **score** |
| --- | --- | --- |
| **MMP23** | Cysteine-switch metalloprotease; roles in reproduction and immunity | −343.38 |
| **MMP25** | MT6-MMP; leukocyte surface protease in inflammation | −330.80 |
| **MMP28** | Epilysin; implicated in skin wound repair | −318.35 |
| **MMP26** | Matrilysin-2; promotes epithelial tumour invasion | −311.04 |
| **MMP4** | Stromelysin-2-like; broad ECM turnover | −304.84 |
| **MMP20** | Enamelysin; essential for tooth enamel formation | −299.28 |
| **MMP11** | Stromelysin-3-like; involved in adipocyte differentiation | −293.55 |
| **MMP6** | Gelatinase-C; basement membrane remodelling | −293.52 |
| **MMP17** | MT4-MMP; GPI-anchored pericellular protease | −287.63 |
| **MMP12** | Macrophage elastase; degrades elastin in inflammation | −287.30 |
| **MMP9** | Gelatinase B; key for leukocyte migration and angiogenesis | −284.87 |
| **MMP21** | Developmental protease; roles in apoptosis and tissue  morphogenesis | −281.75 |
| **MMP15** | MT2-MMP; membrane-bound activator of other pro-MMPs | −277.91 |
| **MMP24** | MT5-MMP; membrane-type with restricted ECM substrate range | −273.84 |
| **MMP19** | Broad-spectrum ECM remodeller; tissue homeostasis | −272.69 |
| **MMP8** | Neutrophil collagenase; mediates inflammatory collagen turnover | −272.30 |
| **MMP5** | Stromelysin-3-like; implicated in tumour progression | −272.11 |
| **MMP27** | Small secreted MMP; possible intracellular retention | −263.74 |
| **MMP22** | Poorly characterized; likely general ECM turnover | −260.91 |
| **MMP7** | Matrilysin; degrades proteoglycans and elastin in tissue repair | −252.41 |
| **MMP16** | MT3-MMP; membrane-bound pericellular protease | −247.55 |
| **MMP1** | Interstitial collagenase; initiates fibrillar collagen degradation | −245.56 |
| **MMP13** | Collagenase-3; central to bone remodelling | −242.51 |
| **MMP10** | Stromelysin-2; degrades a broad range of ECM components | −241.20 |
| **MMP14** | MT1-MMP; pericellular collagenase, key pro-MMP2 activator | −237.56 |
| **MMP18** | Orphan MMP; presumed role in ECM maintenance | −232.90 |
| **MMP3** | Stromelysin-1; activates other MMPs, degrades proteoglycans | −227.91 |
| **MMP2** | Gelatinase A; degrades type IV collagen in basement membranes | −214.97 |

***Note:** MMPs are sorted in ascending order based on docking score computed through HDOCK tool. Selected binding positions were evaluated only if they maintained some contact with SPOCK1 MMP-binding domain.

This table lists MMPs and their respective binding scores to the unique MMP-binding domain of testican-1 (SPOCK1), as computed using the HDOCK docking tool. The MMPs are ranked in ascending order of binding score, with more negative values indicating stronger predicted interactions. Only binding conformations maintaining contact with the testican-1 (SPOCK1) interface were considered. Functional annotations for each MMP are also provided.

**Table S3.** Summary of pro-MMP forms audited against Uniprot and HUGO.

| Panel label used | HGNC symbol | UniProt accession (human) | PDB / model (AlphaFold) | Chain | Species | Structural state | Notes |
| --- | --- | --- | --- | --- | --- | --- | --- |
| MMP1 | MMP1 | **P03956** | AlphaFold model (UniProt P03956) | A | *Homo sapiens* | Zymogen (full-length AF model) | Canonical collagenase |
| MMP2 | MMP2 | **P08253** | AlphaFold model (UniProt P08253) | A | *Homo sapiens* | Zymogen (full-length AF model) | Gelatinase A |
| MMP3 | MMP3 | **P08254** | AlphaFold model (UniProt P08254) | A | *Homo sapiens* | Zymogen (full-length AF model) | Stromelysin-1 |
| MMP7 | MMP7 | **P09237** | AlphaFold model (UniProt P09237) | A | *Homo sapiens* | Zymogen (full-length AF model) | Matrilysin |
| MMP8 | MMP8 | **P22894** | AlphaFold model (UniProt P22894) | A | *Homo sapiens* | Zymogen (full-length AF model) | Neutrophil collagenase |
| MMP9 | MMP9 | **P14780** | AlphaFold model (UniProt P14780) | A | *Homo sapiens* | Zymogen (full-length AF model) | Gelatinase B |
| MMP10 | MMP10 | **P09238** | AlphaFold model (UniProt P09238) | A | *Homo sapiens* | Zymogen (full-length AF model) | Stromelysin-2 |
| MMP11 | MMP11 | **P24347** | AlphaFold model (UniProt P24347) | A | *Homo sapiens* | Zymogen (full-length AF model) | Stromelysin-3 |
| MMP12 | MMP12 | **P39900** | AlphaFold model (UniProt P39900) | A | *Homo sapiens* | Zymogen (full-length AF model) | Macrophage metalloelastase |
| MMP13 | MMP13 | **P45452** | AlphaFold model (UniProt P45452) | A | *Homo sapiens* | Zymogen (full-length AF model) | Collagenase-3 |
| MMP14 | MMP14 | **P50281** | AlphaFold model (UniProt P50281) | A | *Homo sapiens* | Zymogen / membrane-type (AF full model includes pro-/TM region) | MT1-MMP |
| MMP15 | MMP15 | **P51511** | AlphaFold model (UniProt P51511) | A | *Homo sapiens* | Zymogen / membrane-type (AF full model) | MT2-MMP |
| MMP16 | MMP16 | **P51512** | AlphaFold model (UniProt P51512) | A | *Homo sapiens* | Zymogen / membrane-type (AF full model) | MT3-MMP |
| MMP17 | MMP17 | **Q9ULZ9** | AlphaFold model (UniProt Q9ULZ9) | A | *Homo sapiens* | Zymogen / membrane-type (AF full model) | MT4-MMP |
| MMP19 | MMP19 | **Q99542** | AlphaFold model (UniProt Q99542) | A | *Homo sapiens* | Zymogen (full-length AF model) |  |
| MMP20 | MMP20 | **O60882** | AlphaFold model (UniProt O60882) | A | *Homo sapiens* | Zymogen (full-length AF model) | Enamelysin |
| MMP21 | MMP21 | **Q8N119** | AlphaFold model (UniProt Q8N119) | A | *Homo sapiens* | Zymogen (full-length AF model) |  |
| MMP23B | MMP23B | **O75900** | AlphaFold model (UniProt O75900) | A | *Homo sapiens* | Zymogen (full-length AF model) | Note: MMP23 has historical synonyms. |
| MMP24 | MMP24 | **Q9Y5R2** | AlphaFold model (UniProt Q9Y5R2) | A | *Homo sapiens* | Zymogen / membrane-type (AF full model) | MT5-MMP |
| MMP25 | MMP25 | **Q9NPA2** | AlphaFold model (UniProt Q9NPA2) | A | *Homo sapiens* | Zymogen / membrane-type (AF full model) | MT6-MMP |
| MMP26 | MMP26 | **Q9NRE1** | AlphaFold model (UniProt Q9NRE1) | A | *Homo sapiens* | Zymogen (full-length AF model) | Matrilysin-2 / endometase |
| MMP27 | MMP27 | **Q9H306** | AlphaFold model (UniProt Q9H306) | A | *Homo sapiens* | Zymogen (full-length AF model) | N/A |
| MMP28 | MMP28 | **Q9H239** | AlphaFold model (UniProt Q9H239) | A | *Homo sapiens* | Zymogen (full-length AF model) | Epilysin |

*PDB experimental structures were also used where available. This table is for further information of the authors to provide additional context for MMP Zymogen forms in humans.
